# Supplementary figures and images for: Expression of the IL-18-related gene PTX3 correlates with clinicopathological features and prognosis in glioma patients
Source: PeerJ. 2025 Jul 10;13:e19675. doi: 10.7717/peerj.19675 (PMC12256040; doi:10.7717/peerj.19675)

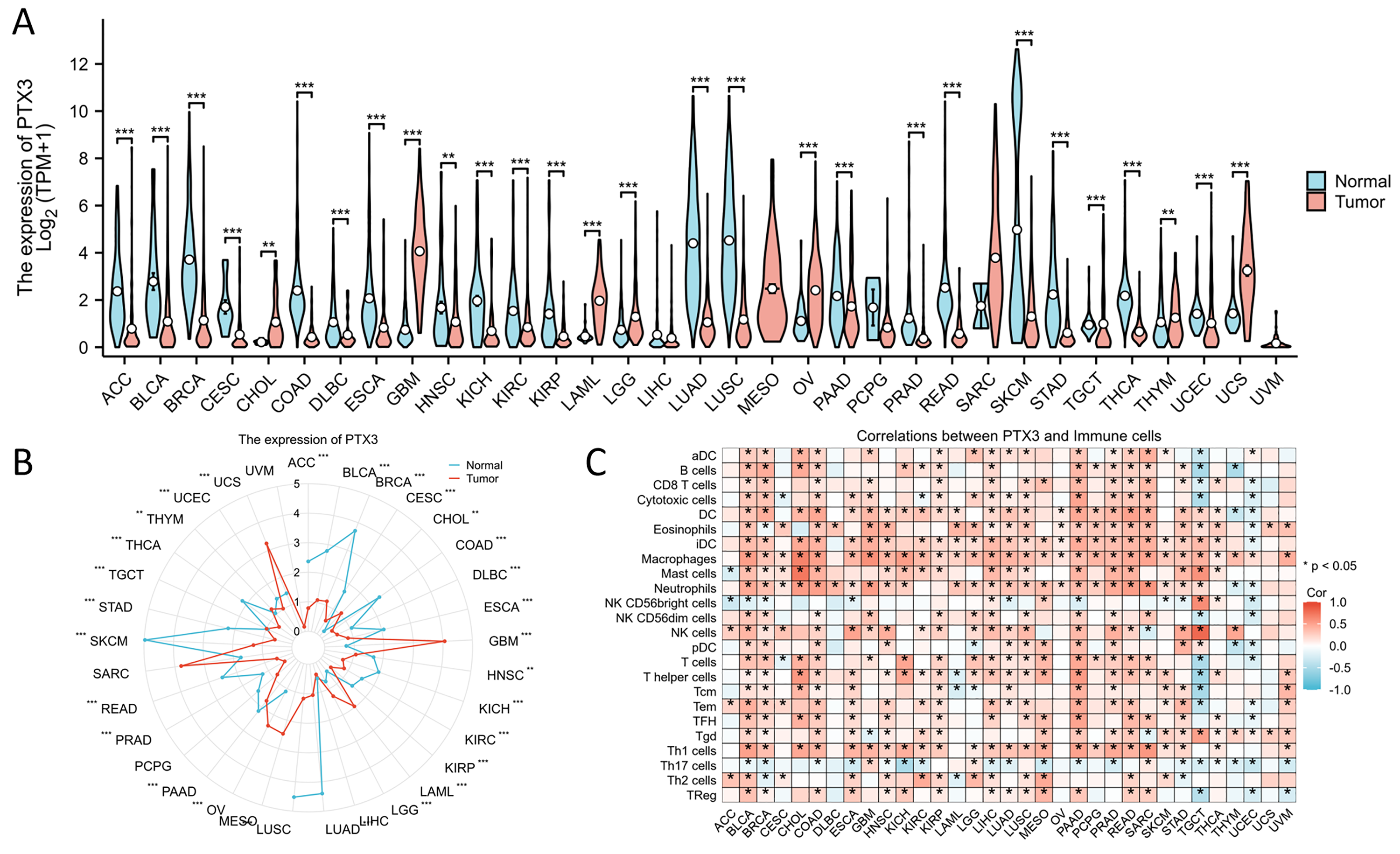

Supplement: Supplemental Information 3 — (A) The comparative analysis of PTX3 expression levels in normal tissues versus various pan-cancer samples. (B) The radar chart illustrates the relative expression levels of PTX3 in different human cancerous tissues when juxtaposed with normal tissue counterparts. (C) The relationship between PTX3 expression and immune cell infiltration across 33 distinct cancer tissue types. *P < 0.05; **P < 0.01; ***P < 0.001. [file peerj-13-19675-s003.png]
