# Supplementary material for: Expression of the IL-18-related gene PTX3 correlates with clinicopathological features and prognosis in glioma patients
Source: PeerJ. 2025 Jul 10;13:e19675. doi: 10.7717/peerj.19675 (PMC12256040; doi:10.7717/peerj.19675)
Supplement: Supplemental Information 4 [file peerj-13-19675-s004.docx]

dat = data.table::fread("gtex_RSEM_gene_fpkm.gz",data.table = F)

library(tidyverse)

exp = column_to_rownames(dat,"sample") %>% as.matrix()

rownames(exp)=rownames(exp)%>%str_split("\\.",simplify=T)%>%.[,1]

library(AnnoProbe)

library(tinyarray)

an=annoGene(rownames(exp),ID_type="ENSEMBL")

exp = trans_array(exp,ids = an,from ="ENSEMBL",to = "SYMBOL")

data_cl=data.table::fread("GTEX_phenotype.gz",header = T,sep ='\t',data.table = F)

data_cl=data_cl[,c(1,3)]

names(data_cl)=c('Barcode','Tissue')

data_cl=data_cl[data_cl$Tissue =='Brain',]

data_cl=data_cl[data_cl$Barcode%in%colnames(dat),]

gtex=exp[,data_cl$Barcode]

rm(an,dat,data_cl1,exp)

y = read.table("TCGA-GBMLGG.htseq_fpkm.tsv.gz",

header = T,

check.names = F,

row.names=1)

y[1:4,1:4]

group4 = make_tcga_group(y);table(group4)

y = trans_exp(y)

y = y[rowSums(y)>0,]

y[1:4,1:4]

yz1=which(grepl("01",str_sub(colnames(y),14,15)))

yz2=which(grepl("06",str_sub(colnames(y),14,15)))

yz=c(yz1,yz2)

yzz=y[,yz]

yc=which(grepl("11",str_sub(colnames(y),14,15)))

ycc=y[,yc]

y=cbind(yzz,ycc)

a=rownames(gtex)[rownames(gtex)%in%rownames(y)]

gtex=gtex[a,]

exp=cbind(y,gtex)

Figure1

library("msigdbr")

m_df = msigdbr(species = "Homo sapiens", category = "C2")

a=which(grepl("WP_IL18_SIGNALING_PATHWAY",

m_df$gs_name))

m1=m_df[a,]

IL18gene=m1$gene_symbol

IL18gene=unique(IL18gene)[table(IL18gene) == 1]

cl = rio::import("TCGA-GBMLGG.survival.tsv")

colnames(cl)[c(2,4)] = c("event","time")

colnames(cl)[2]="event"

colnames(cl)[4]="time"

rownames(cl)=cl$sample

cl=cl[colnames(exp),]

table(rownames(cl)==colnames(exp))

cl=cl[,c(1:4)]

ph = readr::read_tsv("TCGA-GBMLGG.GDC_phenotype.tsv.gz")

ph = ph[,c("submitter_id.samples",

"age_at_initial_pathologic_diagnosis",)]

colnames(ph) = c("sample_id","age")

cl= merge(cl,ph,by.x = "sample",by.y = "sample_id")

exp=exp[,cl$sample]

library(tinyarray)

ec = match_exp_cl(exp[IL18gene,group == "tumor"],

cl,

id_column = "_PATIENT")

cl_matched = ec$cl_matched

exp_matched = ec$exp_matched

x=t(exp_matched)

library(survival)

y = data.matrix(Surv(cl_matched$time,cl_matched$event))

library(glmnet)

set.seed(1000)

cvfit = cv.glmnet(x,

Surv(cl_matched$time,cl_matched$event),

nfold=10, family = "cox")

plot(cvfit)

cl_matched$riskscore = as.numeric(predict(cvfit,newx = x,s = cvfit$lambda.min))

cvfit = cv.glmnet(x, yy, family="cox")

fit=glmnet(x, y, family = "cox")

plot(fit,xvar="lambda",label = T)

library(plotmo)

plot_glmnet(fit)

coef = coef(fit, s = cvfit$lambda.min)

actCoef = coef[coef[,1] != 0,]

lassoGene = row.names(coef)[coef[,1] != 0]

expL=exp[lassoGene,]

expL=cbind(cl,expL)

for(i in colnames(expL[,3:ncol(expL)])){

unicox <- coxph(Surv(time = os_time, event = os_status) ~ df[,i], data = df)

unisum<- summary(unicox)

pvalue <- round(unisum$coefficients[,5],3)

if(pvalue<pfilter){

uniresult <- rbind(uniresult,

cbind(gene=i,

HR=unisum$coefficients[,2],

L95CI=unisum$conf.int[,3],

H95CI=unisum$conf.int[,4],

pvalue=unisum$coefficients[,5]))}}

Figure2

library(corrplot)

M<-cor(t(expL))

library(paletteer)

my_color = rev(paletteer_d("RColorBrewer::RdYlBu"))

my_color = colorRampPalette(my_color)(10)

corrplot(M, type="upper",

method="pie",

order="hclust",

col=my_color,

tl.col="black",

#tl.pos = "d",

tl.srt=45)

library(patchwork)

exp_matched=t(expL)

cl_matched=expL[,c(1,2)]

library(tinyarray)

km_plots = exp_surv(exp_matched,cl_matched)

wrap_plots(km_plots,nrow = 2)

Figure4

exp1=data.frame(t(y))

expn=exp1[order(exp1$"PTX3"),]

PTX3=expn[,"PTX3"]

group = c(rep("low",times=351),

rep("high",times=351))

group = factor(group,

levels = c("low","high"))

expn=t(expn)

library(limma)

design=model.matrix(~group)

fit=lmFit(expn,design)

fit=eBayes(fit)

deg=topTable(fit,coef=2,number = Inf)

library(dplyr)

deg <- mutate(deg,probe_id=rownames(deg))

head(deg)

logFC_t=2

P.Value_t = 0.05

k1 = (deg$P.Value < P.Value_t)&(deg$logFC < -logFC_t)

k2 = (deg$P.Value < P.Value_t)&(deg$logFC > logFC_t)

deg <- mutate(deg,change = ifelse(k1,"down",ifelse(k2,"up","stable")))

table(deg$change)

rownames(deg)=deg$probe_id

deg1=which(grepl("down",deg$change))

deg2=which(grepl("up",deg$change))

deg11=deg$probe_id[deg1]

deg22=deg$probe_id[deg2]

deggenes=c(deg11,deg22)

write.table(deggenes,

file="diffgene.txt",

row.names = F,

col.names = F,

quote = F)

p = deg[deg$change != "stable",

c("symbol","logFC")]

write.table(p,

file = "deg.txt",

sep = "\t",

quote = F,

row.names = F)

Figure5

library(clusterProfiler)

library(org.Hs.eg.db)

ego <- enrichGO(gene = deggenes,

OrgDb = org.Hs.eg.db,

pvalueCutoff =0.05,

qvalueCutoff =0.05,

readable = TRUE)

head(ego)

write.table(ego,file="GO_result.txt",sep="\t",

quote=F,row.names = F)

kk <- enrichKEGG(gene = deggenes,

organism = "hsa",

pvalueCutoff =0.05,

qvalueCutoff =1)

head(kk)

write.table(kk,file="KEGG_result.txt",sep="\t",quote=F,row.names = F)

Figure6

library(org.Hs.eg.db)

library(clusterProfiler)

library(enrichplot)

genelist_input <-deg[,c(1,6)]

genename <- as.character(genelist_input[,1])

gene_map <- select(org.Hs.eg.db, keys=genename, keytype="SYMBOL", columns=c("ENTREZID"))

colnames(gene_map)[1]<-"Gene"

write.csv(as.data.frame(gene_map)

aaa<-inner_join(gene_map,genelist_input,by = "Gene")

aaa<-aaa[,-1]

aaa<-na.omit(aaa)

aaa$logFC<-sort(aaa$logFC,decreasing = T)

geneList = aaa[,2]

names(geneList) = as.character(aaa[,1])

geneList

Go_gseresult <- gseGO(geneList, 'org.Hs.eg.db', keyType = "ENTREZID", ont="all", nPerm = 1000, minGSSize = 10, maxGSSize = 1000, pvalueCutoff=1)

KEGG_gseresult <- gseKEGG(geneList, nPerm = 1000, minGSSize = 10, maxGSSize = 1000, pvalueCutoff=1)

Go_Reactomeresult <- gsePathway(geneList, nPerm = 1000, minGSSize = 10, maxGSSize = 1000, pvalueCutoff=1)

gseaplot2(Go_Reactomeresult,212,pvalue_table = TRUE)

Figure7

library(GSVA)

gene_set<-read.csv("mmc3.csv",header=T,stringsAsFactors = FALSE)

list<- split(as.matrix(gene_set)[,1], gene_set[,2])

gsva_matrix<-gsva(as.matrix(expn), list,method='ssgsea',kcdf='Gaussian',abs.ranking=TRUE)

row.names(group)=group$sample

group=group[colnames(expn),]

expn=as.data.frame(t(expn))

expn$cluster=group$Group

expn=expn[,c(29,1:28)]

test=as.list(expn[,-1])

height<-stack(test)

Group=rep(expn$cluster,ncol(expn)-1)

data1=as.data.frame(cbind(Group,height))

library(ggplot2)

library(ggpubr)

library(ggsignif)

colnames(data1)=c("group","Cell_infiltration_level","cell")

df=data1

p=ggplot(data=df,aes(x=cell,y=Cell_infiltration_level,fill=group))+

geom_boxplot(width=0.3,

position = position_dodge(0.5),outlier.colour =NA)+

theme_bw()+

theme(panel.grid = element_blank())+

scale_fill_manual(values = c("#4DBBD5FF","#E64B35FF","#E18727FF"))+

stat_compare_means(aes(group=group),

label="p.signif",

hide.ns = TRUE,

label.y = c(0.6))+

theme(axis.text.x = element_text(size=12,colour="black",angle=60,hjust = 0.9),axis.text.y = element_text(size=12, angle=0),

axis.title.x = element_text(size=15),axis.title.y = element_text(size=15))

Figure8

exp<-read.table("GSE50161_series_matrix.txt.gz",sep="\t",quote = "",

fill=T,comment.char = "!",header=T)

rownames(exp)<-exp[,1]

exp<-exp[,-1]

expn=exp[,103:115]

expt=exp[,47:80]

exp=cbind(expt,expn)

Group = c(rep("Tumour",times=34),

rep("Normal",times=13))

Group = factor(Group,

levels = c("Normal","Tumour"))

table(Group)

library(tinyarray)

find_anno('GPL570')

ids <- AnnoProbe::idmap('GPL570')

library(dplyr)

library(stringr)

exp=as.data.frame(exp)

rownames(exp)=substr(rownames(exp),2,nchar(rownames(exp)))

rownames(exp)=str_sub(rownames(exp),1,nchar(rownames(exp))-1)

exp$probe_id=rownames(exp)

table(exp$probe_id%in%ids$probe_id)

exp2= merge(exp,ids,by.x="probe_id", by.y="probe_id")

exp2=exp2[!duplicated(exp2$symbol),]

rownames(exp2)=exp2$symbol

exp=exp2

which(grepl("PTX3",exp$symbol))

expPTX3=exp[5342,]

path<-file.path("D:","ff.xls")

library(xlsx)

write.xlsx(expPTX3,file = path)

exp<-read.table("GSE7696_series_matrix.txt.gz",sep="\t",quote = "",

fill=T,comment.char = "!",header=T)

rownames(exp)<-exp[,1]

exp<-exp[,-1]

which(grepl("206157_at",rownames(exp)))

expPTX3=exp[15604,]

path<-file.path("D:","ff.xls")

library(xlsx)

write.xlsx(expPTX3,file = path)

library(dplyr)

library(ggplot2)

dat = deg[!duplicated(deg$probe_id),]

p <- ggplot(data = dat,

aes(x = logFC,

y = -log10(P.Value))) +

geom_point(alpha=0.4, size=3.5,

aes(color=change)) +

ylab("-log10(Pvalue)")+

scale_color_manual(values=c("blue", "grey","red"))+

geom_vline(xintercept=c(-logFC_t,logFC_t),lty=4,col="black",lwd=0.8) +

geom_hline(yintercept = -log10(P.Value_t),lty=4,col="black",lwd=0.8) +

theme_bw()

p

for_label <- dat%>%

filter(probe_id %in% c(“PTX3"))

volcano_plot <- p +

geom_point(size = 3, shape = 1, data = for_label) +

ggrepel::geom_label_repel(

aes(label = probe_id),

data = for_label,

color="black"

)

volcano_plot

Figure11

library(regplot)

library(survival)

mod <- coxph(formula = s,data=cl_matched)

regplot(mod,

observation=cl_matched[1,],

obscol = "#326db1",

failtime = c(1095,1825),

plots = c("no plot","no plot"),

points = T,

prfail = T)

library(rms)

options(datadist=datadist(dat))

f3 <- cph(formula = s,

data=dat,x=T,y=T,surv = T, time.inc=1095)

cal3 <- calibrate(f3, cmethod="KM", method="boot", u=1095, m=50, B=1000)

f5 <- cph(formula = s,

data=dat,x=T,y=T,surv = T, time.inc=1825)

cal5 <- calibrate(f5, cmethod="KM", method="boot", u=1825, m=50, B=1000)

png("plot/cal.png")

plot(cal3,lwd = 2,lty = 0,errbar.col = c("#2166AC"),

bty = "l",

xlim = c(0,1),ylim= c(0,1),

xlab = "Nomogram-prediced OS (%)",ylab = "Observed OS (%)",

col = c("#2166AC"),

cex.lab=1.2,cex.axis=1, cex.main=1.2, cex.sub=0.6)

lines(cal3[,c('mean.predicted',"KM")],

type = 'b', lwd = 1, col = c("#2166AC"), pch = 16)

mtext("")

plot(cal5,lwd = 2,lty = 0,errbar.col = c("#B2182B"),

xlim = c(0,1),ylim= c(0,1),col = c("#B2182B"),add = T)

lines(cal5[,c('mean.predicted',"KM")],

type = 'b', lwd = 1, col = c("#B2182B"), pch = 16)

abline(0,1, lwd = 2, lty = 3, col = c("#224444"))

legend("topleft",

legend = c("1-year","3-year","5-year"),

col =c("#2166AC","#B2182B"),

lwd = 2,

cex = 1.2,

bty = "n")
